# Tracking and debriefing birth data at scale: A mobile phone application to improve obstetric and neonatal care in Bihar, India

**DOI:** 10.1002/nop2.134

**Published:** 2018-03-12

**Authors:** Hilary Spindler, Jessica Dyer, Kingshuk Bagchi, Vikash Ranjan, Amelia Christmas, Susanna R. Cohen, Mona Sterling, Malay Bharat Shah, Aritra Das, Tanmay Mahapatra, Dilys Walker

**Affiliations:** ^1^ Global Health Sciences University of California San Francisco San Francisco CA USA; ^2^ Pronto International Seattle Seattle WA USA; ^3^ CARE India Solutions for Sustainable Development Bihar Technical Support Unit Patna Bihar India; ^4^ Pronto International State RMNCH+A Unit Patna Bihar, India; ^5^ College of Nursing University of Utah Salt Lake City UT USA; ^6^ Department of Obstetrics and Gynecology and Reproductive Services University of California San Francisco San Francisco CA USA

**Keywords:** India, maternal care, midwife, neonatal care, nurse

## Abstract

**Aim:**

This analysis assessed changes over time in skill and knowledge related to the use of evidence‐based practices associated with quality of maternal and neonatal care during a nurse midwife mentoring intervention at primary health clinics (PHCs) in Bihar, India.

**Design:**

Nurse midwife mentors (NMMs) entered live birth observation data into a mobile App from 320 PHCs.

**Methods:**

The NMMs completed prompted questions in the App after every live birth witnessed. The App consisted of questions around three main themes, “What went well?”, “What needed improvement?” and “What can be done differently next time?”.

**Results:**

Observational data from 5,799 births was recorded by 120 NMMs in 320 PHCs. Knowledge and skill during normal spontaneous vaginal deliveries and complicated deliveries with either a postpartum haemorrhage or non‐vigorous infant all showed statistically significant improvement (*p *<* *.001) over time using a Chi‐squared test for trend with a mean increase of 41% across all indicators.

## 1 INTRODUCTION

Despite substantial reduction in neonatal and maternal mortality over past decades, India remains the largest contributor in the global burden of neonatal deaths with 700,000 annual deaths among neonates (UNICEF et al., 2014; World Bank, 2015). Bihar, a state in north eastern India, is one of the poorest and most populated in the country (UNICEF, 2014). In Bihar, both the maternal and neonatal mortality ratios are high at an estimated 208 (163–253) deaths per 100,000 live births for mothers and a reported 27 deaths per 1,000 live births for neonates (Office of the Registrar General & Census Commissioner India, 2014). Available evidence suggests that the quality improvement of obstetric and newborn care, especially management practices during birth, could prevent a proportion of these deaths (Bhutta, Darmstadt, Hasan, & Haws, 2005; Campbell & Graham, 2006; Goudar et al., 2015; Iyengar et al., 2014; Pasha et al., 2010).

To address this high level of maternal and neonatal mortality, a large quality of care improvement initiative was implemented in Bihar by the non‐governmental organization, CARE India, in close collaboration with the State Government of Bihar (Das et al., 2016). The state‐wide initiative aimed to improve the quality of obstetric and neonatal clinical care provided at every primary health clinic (PHC) in the state of Bihar through a variety of interventions including infrastructure improvements, increased supply procurement and the implementation of a mobile nurse‐mentoring programme. The mobile nurse‐mentoring programme aimed to improve auxiliary nurse midwife (ANM) and general nurse midwife (GNM) clinical skill and management practices during birth. To accomplish this, PRONTO International (PRONTO) partnered with CARE India to integrate simulation, team training and postevent debriefing after live births into the nurse‐mentoring programme. Postevent debriefing, is understood to be an effective aspect of clinical education, quality improvement and systems learning (Agency for Healthcare Research and Quality, 2016) as it provides a space for self‐reflection by providers including their role and behaviour, knowledge and skills, and team operation. In this intervention, postevent debriefing of live births was defined as a “structured and guided reflection process where students actively appraised their cognitive, affective and psychomotor performance within the context of their clinical judgment skill” (Al Sabei & Lasater, 2016).

To our knowledge, no studies in Bihar have been conducted measuring changes in clinical knowledge and skill using data from postevent debriefs after live births. Knowledge and skill indicators from postevent debriefs of live births were collected using a mobile Application (App) as part of the nurse‐mentoring programme. The mobile App was designed as a job aid to lead guided debriefs with clinical site staff based on observations recorded during live births. Mobile Apps have been previously shown in pilot studies to have potential benefits to debriefing in advanced life support simulations such as enabling the debriefer to have a more intuitive visual summary of the skills and techniques used during practice (Chang, Su, Lin, & Huang, 2015). Debriefers who used the App, were found to provide more and richer feedback than those not using an App (Chang et al., 2015). Additionally, studies evaluating programme outcomes of community health workers in low‐resource settings have provided some evidence that mobile tools can help to improve the quality of care provided (Braun, Catalani, Wimbush, & Israelski, 2013). This paper discusses the results from the implementation of a postevent debriefing intervention by looking at the feasibility of a mobile App as both a job aid for postevent debriefing as well as to track changes in provider skill, teamwork and supply availability overtime.

In this study of a cross‐sectional programmatic intervention, we examined the changes over time in ANM/GNM clinical knowledge and skill during deliveries at PHCs in Bihar, India. The findings were based on data collected during postevent debriefs during the nurse‐mentoring intervention. This primary aim of our study was to assess the effectiveness of the nurse‐mentoring programme in improving quality of ANM/GNM clinical skill and knowledge during birth. The secondary aim of our study was to assess the acceptability of integrating a mobile App as a job aid during deliveries at PHCs in Bihar.

## 2 METHODS

### 2.1 Design

CARE India, working closely with the Government of Bihar, implemented the Integrated Family Health Initiative project in 2011 aimed at the reduction in maternal and childhood mortality in eight priority districts in Bihar (CARE India, 2013). The initiative was subsequently scaled up to all 38 districts in the state of Bihar beginning in January 2015 and ending in January 2017. As a part of the subsequent state‐wide implementation of the programme, mobile nurse mentoring was conducted using a phased approach to roll‐out over the course of 8 months in four phases. Each phase targeted 80 PHC facilities which provided basic emergency obstetric and neonatal care. PHCs were assigned to phases based on delivery load, facility readiness and support among leadership. By the end of the fourth phase, 320 PHC facilities in Bihar had received the nurse‐mentoring programme. In the PHCs, mentorship was provided to six to eight ANM/GNMs working at each facility. ANM/GNMs were selected for participation in the programme based on having labour room duties as well as their interest in participation.

During implementation of the four phases, 120 nurse midwife mentors (NMMs) worked in pairs spending 1 week per month at each of their four assigned facilities, constituting eight total weeks of on‐site mentorship per PHC state wide. On‐site mentoring included an 8‐week curriculum. The activities and inputs of this curriculum included simulation training, teamwork training, skills building and postevent debriefing after live births. Each of the four phases of implementation had the same curriculum. Overall, the NMMs were younger and less clinically experienced but had higher levels of clinical education than the ANM/GNM site staff or “mentees” they were working with at the PHCs. Most NMMs were not from the state of Bihar and had relocated temporarily for the job.

### 2.2 Method

Use of postevent debriefing of live births as a mentoring strategy was implemented in PHCs. In the first phase of the programme, the debriefing tool was piloted as a paper‐based tool in 80 PHCs. For the next three phases the debriefing tool was implemented using a mobile App platform in 240 PHCs. Each NMM underwent a 1‐hr training on how to use the mobile App with a practice session for troubleshooting prior to launch in addition to practical training on postevent debriefing. The Qualtrics Offline mobile App (https://www.qualtrics.com/) was initially used during the second phase. However, after NMMs reported a series of technical glitches, the offline mobile App data collection was migrated to the free and open‐source OpenDataKit (ODK) platform (https://opendatakit.org/) which was developed and tested by the Database Management System development team of CARE India. The third and fourth phases of the nurse midwife mentoring continued to use the ODK platform. For this analysis only data collected on the mobile App platform from 2015‐2017 (Qualtrics in Phase 2 and ODK in Phases 3–4) was included. Data from Phase 1 were excluded as some NMMs reported that the paper‐based format was confusing and difficult to understand.

The NMMs were instructed to complete the prompted questions in the mobile App for every live birth they witnessed during their time at a facility. The NMMs completed the form either as they were observing the birth or immediately afterwards. The mobile App consisted of questions focused around three main themes, “What went well?”, “What needed improvement?” and “What can be done differently next time?”. The responses to “What went well?” stratified by different birth scenarios based on complications were used as the outcome for our study and served as an indication of changes in the quality of ANM/GNM clinical skill and knowledge over time during the mentoring intervention. Categories included evidence‐based clinical practice, teamwork and communication, and supply availability. The mobile App also included questions on the use of specific evidence‐based clinical practices predefined by a project team of medical experts as required best practices for each normal birth (i.e. 10 International Units of Oxytocin administered postdelivery, controlled cord traction performed, uterine tone assessed, infant dried and stimulated immediately, infant placed on abdomen, delayed cord clamping performed, breastfeeding initiated, uterine massage conducted, blood pressure taken and reported, pulse taken and reported, and family planning offered and performed). Additionally, Team STEPPS^TM^ teamwork and communication techniques were introduced through the intervention and measured through questions on the mobile App (i.e. think‐out‐loud (transparent‐ thinking), SBAR, check back, call out, mother spoken to directly, mother spoken to kindly, call for help, help arrived and transfer plans initiated). Supply availability based on visual verification was also collected through questions in the mobile App (i.e. antiseptic solution, sterile gloves, clean towel, sterile ties/clamps, sterile blade, Oxytocin, intravenous (IV) supplies, ambu‐bag and mask and suction device). Questions around “what went well” vs. “what needed improvement” were presented in a dichotomous format requiring the NMM to provide a subjective assessment based on their observation of how the mentored providers performed during the observed birth. A response to each question was required to move on to the next question and there was also an option to mark “Not applicable” if a question was not relevant in a specific case and “Don't know.” These responses were excluded from the analysis.

The mobile App asked if a complication occurred during the observed birth and if so a series of topic‐specific questions were then generated to supplement those that appeared for every birth around the additional agreed on evidence‐based practices. The four complication types that generated follow‐up questions in the mobile App were postpartum haemorrhage, non‐vigorous infant, pre‐eclampsia and preterm birth. Postpartum haemorrhage was defined as abnormal blood loss estimated above 500 ml. A non‐vigorous infant was defined as a baby born without crying and asphyxia referred to a baby born without crying after drying and stimulation were performed. Pre‐eclampsia was defined as onset blood pressure of greater than or equal to 140/90 after 20 weeks of gestation, with or without features of severe pre‐eclampsia. Preterm birth was defined as the birth of a baby at gestational age of less than 37 weeks 0 days. The indicators collected for these complications included: pre‐eclampsia (identification of hypertension, identification of hyperreflexia, identification of pre‐eclampsia, reflexes checked, magnesium given), preterm (diagnosed as preterm labour before delivery, identified as preterm baby, IV placed, steroids given, tocolytics given), non‐vigorous infant (immediate newborn care provided, identification of non‐vigorous infant, identification of asphyxia, identification of meconium, bag and mask ventilation, suctioning), postpartum haemorrhage (identification of abnormal bleeding, 20 IU of oxytocin given and uterotonics given). NMM were also able to select “Other” for a complication not listed and instructed to input the type into a text field. This response, however, did not generate additional questions in the mobile App.

When the NMM stepped in to either assist or conduct the delivery themselves, based on their ethical obligation to the patient, they were still instructed to answer the mobile App questions based on their opinion of how the “mentees” performed or would have performed, not on how they actually performed with assistance. NMM's were also asked in the mobile App about the level of support they provided during the delivery to the mentees. This was included to have an indication of level of assistance that was received during each birth.

The final page of the mobile App was a customized screen that provided the NMM with a tailored debriefing guide specific to the birth they had just observed based on the data they had entered. The feedback guide was designed to aid the NMMs in facilitating a debrief with the participating providers.

Additionally, to better understand both acceptability and use patterns of the mobile App, in August 2016, the NMMs from Phase 4 were asked to complete a brief five‐question online anonymous survey about the mobile App.

### 2.3 Analysis

Aggregate level data collected on the ODK mobile App platform was available in the format of pre‐specified tables and figures that could be filtered by date and mentorship phase for viewing on an online ONA dashboard (https://ona.io/home/) available to all NMMs and project staff. The ONA dashboard data were manually updated by the Concurrent Measurement and Learning team of CARE India on a weekly basis.

Data collected using the Qualtrics offline mobile App in the second phase and the ODK mobile App in the third and fourth phases were exported into MS Excel (https://products.office.com/en-us/excel) from the online repository and evaluated for range and logic checks by PRONTO staff based in Patna. Data were then transferred to the University of California San Francisco for further cleaning and analysis using R Studio (https://www.rstudio.com/).

We calculated frequency of the occurrence of each of the knowledge and skill evidence‐based clinical indicators practiced during live births during each month of the mentoring intervention and measured changes over time using the Chi‐squared test for trend and stratified by complication type for indicators only indicated for specific clinical events.

### 2.4 Ethics

The Institutional Review Board committee of the Indian Institute of Health Management Research in Jaipur and the Committee for Human Research at the University of California San Francisco reviewed and approved the study (Study ID# 14‐15446).

## 3 RESULTS

In Phases 2–4, live birth observations were recorded in the mobile App for 4,923 deliveries at the 240 PHC facilities mentored by 120 NMMs (Table 1). Of the observed births, 22% were identified as having one or more complication (*N *=* *1,088).

**Table 1:** Descriptive statistics of debriefing mobile App data collected during live births, Phases 2–4 in Bihar, India

	Phase 2	Phase 3	Phase 4	Total
	21 Sept 2015–12 May 2016	14 Dec 2016–5 July 2016	4 July 2016–31 Jan 2017	21 Sept 2015–31 Jan 2017
# live birth observations	1,303	1,364	2,256	4,923
# facilities	80	80	80	240
% of observed births with 1 or more complications^a^	20% (260)	31% (418)	18% (410)	22% (1088)
Postpartum haemorrhage	5% (70)	11% (156)	4% (87)	6% (313)
Non‐vigorous infant	7% (93)	9% (129)	5% (108)	7% (330)
Meconium present	6% (74)	7% (89)	3% (73)	5% (236)
Preterm birth	2% (26)	2% (23)	2% (41)	2% (90)
Pre‐eclampsia	0% (5)	1% (10)	0% (10)	1% (25)
Other	6% (83)	12% (160)	9% (195)	9% (438)

a Multiple complications can be coded for an individual birth.

Among the births recorded as having one or more complication, having a non‐vigorous infant was the most common type of complication which occurred in 7% of births, followed by postpartum haemorrhage in 6% of births, preterm birth in 2% and pre‐eclampsia in 1%. “Other” complications including, for example, low birth weight, cervical and perineal tears, breech presentation, still‐birth, retained placenta and multiple births were recorded in 6% of births. Additionally, 236 births had two or more complications recorded which accounted for roughly one of every five complicated births (or 1 of every 20 births overall). The most commonly recorded dual complication was a non‐vigorous infant and meconium present which occurred in 11% of all complicated births. Four per cent of complicated births reported a postpartum haemorrhage and a non‐vigorous infant and 2% reported a non‐vigorous and preterm infant.

Based on ethical obligations at times when NMMs observed a complication and assistance was required they stepped in to assist with the delivery. Their level of participation was assessed on a Likert scale from “1” meaning no assistance to “10” indicating the NMM conducted the entire delivery. During complicated births the score averaged 4.9 compared with 4.2 in normal births. Additionally, a question at the end of the mobile App ensured that NMMs could select who “conducted” and who “participated” in each delivery with “NMM” as one of the job classifications. In 77% of complicated cases the NMM reported that they “conducted” the delivery themselves, whereas in normal deliveries the NMM reported that they “conducted” the delivery in 72% of births. Since these high levels of intervention by the NMM's in the deliveries bias the results of whether a clinical practice occurred or not, our analysis focuses only on data collected through the questions where NMM's were asked to subjectively assess the mentees performance by determining “what went well” and “what needed improvement” during the delivery.

Trends over time all showed statistically significant improvement at *p *<* *.001 for all normal spontaneous vaginal delivery, non‐vigorous infant and postpartum haemorrhage related indicators based on a Chi‐squared test for trend. On average, the normal spontaneous vaginal delivery indicators increased by 43% from the first month of mentoring to the last month of mentoring, while the non‐vigorous infant indicators increased by 44% and the postpartum haemorrhage indicators increased by 33%. Tables 2, 3, 4 show changes over time for the subjective data based on the NMM's perception of what “went well” vs. “what needed improvement.”

**Table 2:** Changes over time^a^ in normal spontaneous vaginal deliveries indicators around “what went well” based on debriefing mobile App data collected during live births at 240 Phases 2–4 facilities in Bihar, India (*N* = 4923)

	Week 1	Week 2	Week 3	Week 4	Week 5	Week 6	Week 7	Week 8	*p*‐value^a^
Supply availability	20% (111)	26% (190)	32% (260)	43% (240)	49% (270)	62% (349)	63% (318)	60% (127)	<.001
Personnel/staffing	35% (192)	41% (297)	41% (338)	45% (255)	64% (356)	67% (378)	69% (349)	68% (144)	<.001
Team communication	17% (92)	22% (156)	25% (204)	32% (179)	50% (278)	52% (292)	56% (282)	56% (119)	<.001
Cleanliness	19% (105	22% (162)	28% (228)	42% (238)	56% (309)	62% (349)	56% (281)	50% (105)	<.001
Communication with mother	44% (245)	58% (420)	61% (501	73% (411)	81% (448)	81% (460)	85% (426)	82% (173)	<.001
AMTSL knowledge	40% (221)	64% (467)	70% (577)	80% (450)	88% (488)	90% (507)	92% (466)	88% (186)	<.001
Delayed cord clamping knowledge	44% (241)	68% (492)	71% (588)	78% (438)	88% (487)	89% (502)	88% (445)	85% (181)	<.001
Immediate newborn care knowledge	30% (166)	47% (343)	54% (443)	64% (361)	73% (401)	80% (454)	84% (423)	82% (173)	<.001
Immediate new born care skills	24% (134)	37% (269)	45% (370)	57% (322)	66% (366)	73% (414)	79% (400)	77% (163)	<.001
Vital signs taken	25% (137)	42% (306)	50% (411)	63% (353)	66% (364)	77% (436)	75% (380)	75% (158)	<.001
Vital signs reported/recorded	17% (92)	26% (186)	37% (306)	54% (302)	56% (308)	72% (408)	70% (353)	68% (145)	<.001

a Chi‐squared test of trend.

**Table 3:** Changes over time^a^ in neonatal resuscitation indicators around “what went well” based on debriefing mobile App data collected during live births at 240 Phases 2–4 facilities in Bihar, India (*N* = 330)

Non‐vigorous infant (*n* = 330)	Week 1	Week 2	Week 3	Week 4	Week 5	Week 6	Week 7	Week 8	*p*‐value^a^
Identification of non‐vigorous infant	24% (9)	41% (16)	49% (29)	58% (24)	73% (27)	78% (28)	74% (42)	74% (17)	<.001
Identification of asphyxia	13% (5)	33% (13)	37% (22)	59% (24)	62% (23)	83% (30)	68% (39)	61% (14)	<.001
Identification of meconium	32% (23)	49% (19)	39% (23)	41% (17)	57% (21)	47% (17)	56% (32)	78% (18)	<.001
Knowledge & skills of Bag and mask ventilation	16% (6)	18% (7)	19% (11)	22% (9)	27% (10)	44% (16)	40% (23)	48% (11)	<.001
Suctioning knowledge & skills	26% (10)	31% (12)	37% (22)	54% (22)	70% (26)	75% (27)	60% (34)	70% (16)	<.001

a Chi‐squared test of trend.

**Table 4:** Changes over time^a^ in postpartum haemorrhage indicators around “what went well” based on debriefing mobile App data collected during live births at 240 Phases 2–4 facilities in Bihar, India (*N* = 313)

Postpartum haemorrhage (*n* = 313)	Week 1	Week 2	Week 3	Week 4	Week 5	Week 6	Week 7	Week 8	*p*‐value^a^
Identification of abnormal bleeding	55% (11)	52% (22)	54% (33)	78% (38)	86% (36)	75% (33)	92% (34)	94% (15)	<.001
IV placed	65% (13)	55% (23)	71% (44)	78% (39)	81% (34)	82% (36)	76% (28%)	100% (16)	<.001
20 IU of Oxytocin added in RL	50% (10)	48% (20)	50% (31)	68% (34)	81% (34)	59% (26)	54% (20)	88% (14)	<.001
Knowledge of dose of uterotonic & administration of uterotonic	41% (9)	51% (23)	54% (31)	63% (31)	76% (35)	55% (24)	56% (20)	60% (9)	<.001

a Chi‐squared test of trend.

**Figure 1:**
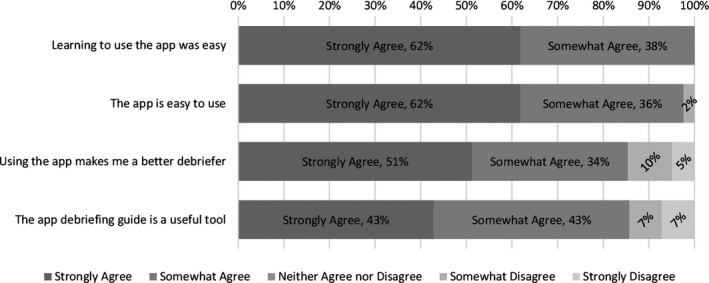
Perceptions of the mobile App based on a anonymous survey data collected from Phase 4 NMMs at facilities in Bihar, India (*N* = 42)

A total of 42 NMMs from Phase 4 responded to the online anonymous survey about the mobile App, which equates to every NMM participating in the phase as well as two additional master mentors charged with programme oversight. Overall, Phase 4 NMMs found the mobile App very easy to learn how to use and useful as a debriefing tool. As shown in Figure 1, 85% of respondents felt that the mobile App made them a better debriefer and 86% of respondents felt that the debriefing guide in the mobile App was a useful tool. Seventy‐four per cent of respondents reported that they used the mobile App during or after deliveries “always” or “most of the time.”

Respondents were asked the question, “What do you like about using the mobile App?” Respondents most commonly stated positive characteristics including the mobile App being easy to use, offline and contributing to debriefs. Two NMMs stated:

“It's easy, quick and helps us cover all that we need to [in the] debrief.”

“It is easy to use and doesn't take much time. It is offline.”

Respondents also appreciated that the mobile App helped them to see the areas where the mentees needed more practice. One respondent stated: “It helps to know in which part mentees need more practice.” Lastly, one respondent appreciated how the data from the mobile App helped her to see improvements over time. She stated that the mobile App “help[ed] in debrief and realized how much we improve[d]”.

Respondents were also asked to respond to the question: “What were the biggest challenges you faced in using the mobile App?”. The most frequently cited challenge with using the mobile App to debrief after live cases was a high facility delivery load (i.e. number of births at the facility). Phase 4 NMMs explained that at their facilities, there was a high number of deliveries occurring each day, thus preventing the completion of the mobile App for actual cases. As two NMMs stated:

“Sometimes we complete the App before debrief and sometimes we are unable to do so because of other work and delivery load.”

“Though it is easy to use but not always possible to use immediately after delivery because of high delivery load in some facilities.”

Other, less commonly mentioned challenges included network and technological problems. While a mobile network was not required to complete the mobile App, a network was required to upload the data to the server. As one respondent stated: “Sometime[s] network is not available in [the] PHC.” Lastly, NMMs mentioned technological problems including the mobile App not working on various operating systems as well as glitches in the mobile App. Two NMMs stated:

“Sometimes the App doesn't work properly.”

“In some mobile it's not supporting.”

## 4 DISCUSSION

We found that the nurse mentorship model is an effective method of transferring basic emergency obstetric and neonatal care knowledge and skills in low‐resource settings. Results show that the mobile App is an acceptable job aid in a nurse‐mentoring programme in Bihar and that it can be used to monitor changes in medical management, teamwork and supply availability over time. Additionally, these data support larger study results, presented elsewhere (Das et al., 2016), that suggest that the larger state‐wide program increases these indicators over time. Other implementation programmes using skills training in India have also pointed to improved provider skill and knowledge (Bellad et al., 2016; Goudar et al., 2013). The uniqueness of this programme is the integration of high‐fidelity simulation in a nurse‐mentoring programme that included bedside mentoring. In this analysis, all indicators monitored for what “went well” showed a statistically significant increase from the first month of the nurse mentorship programme to the final month. With the mobile App's programmatic feedback made available, NMMs were better informed in terms of what knowledge and skill transfers needed further emphasis while supervisors and programme officials could use the data to develop insight into the conditions and practices encountered at the mentored facilities. Additionally, given the large sample size of observed births being entered by many NMM's accompanied by the fact that NMM's could not see the data on an aggregate level after it was entered, we believe these improvements over time are reliable and do not simply reflect a social desirability of the mentors to demonstrate improvement of their mentees. To our knowledge, this is one of the first cross‐sectional studies to examine the effectiveness of the nurse‐mentoring programme in improving quality of ANM/GNM clinical skill and knowledge during deliveries using a mobile App job aid for postevent debriefing in Bihar. We found that 85% of mentors felt that the mobile App made them a better debriefer and 86% felt that the debriefing guide in the mobile App was a useful tool.

We also found that, communication with the mother during delivery was another indicator that showed strong improvement from beginning of the intervention to the end (Table 2). This suggests that gains are an important indication of respectful maternity care (White Ribbon Alliance, 2012). The authors posit that this improvement may be in part due to the use of a female member of the provider team playing the role of the patient in the simulated births done as part of the mentorship training at their PHC. This is done so that the mentees and mentors have an opportunity to intimately experience what it is like to be a patient in the facility. These experiences are then discussed during the simulation debriefing sessions. The person that played the patient is specifically asked to reflect on how it felt to be the patient with the purpose of increasing provider empathy and use of therapeutic communication. Participation such as this may have contributed to the reported change in provider–patient interactions during observed births. Findings from a prospective randomized controlled trial in six United Kingdom hospitals and the Bristol Medical Simulation Center have found that training using a patient‐actor may be better at improving perception of safety and communication than training with a computerized manikin simulator (Crofts et al., 2008). Interestingly, significant improvements around supply availability were also reported during the span of the nurse midwife mentoring programme, although the overarching intervention had no specific arm that directly addressed the issue. This is believed to be one of the unintended, yet beneficial, consequences of this intervention. These improvements were likely due to NMMs and mentees gaining a better understanding through the programmatic curriculum of the inherent importance and need for adequate supplies, including medications, to ensure safe and healthy outcomes for both the mother and baby. It is possible that supplies in a facility were made more readily accessible to facility staff by removing physical barriers to acquiring them (i.e. opening locks on cabinets, etc.) and that PHC patients were increasingly instructed on how to acquire supplies and medications independently as there were asserted, yet unconfirmed, reports of increased medication purchasing by community members.

The routine monitoring of these data from the mobile App was also an effective method for data‐driven monthly programmatic feedback to the NMMs themselves. The feedback enhanced their understanding of how facilities were performing in terms of both clinical knowledge and skills as well as teamwork and communication practices. This iterative feedback loop enabled NMMs to better implement targeted interventions to accelerate site‐level learning at their assigned facilities. For example, mentors could respond to the data by increasing the number of in situ simulations focused around a specific clinical event. Overall, the qualitative survey results around perceptions of the mobile App from Phase 4 NMMs were positive. While NMMs found the mobile App easy to learn and use, the high delivery loads in the facility prevented them from always completing it prior to debriefing. The authors also posit that the NMMs did not always complete the mobile App and subsequent debrief since doing so not only took time but also may have been perceived as redundant since mentors engaged in such a large amount of coaching during the actual deliveries.

### 4.1 Limitation

This study had several limitations. The NMMs did not complete the live witness debrief tool after every birth for which they were present which may have had an impact on the results in terms of the rends over time for the “what went well?” indicators collected. Due to this programmatic limitation, the data collected is a convenience sample of births. The overarching biases around which types of births were more likely to be entered is unknown. It is likely that despite asking for the completion of live‐witnessed debriefs for each of the live observed birth, there may be high likelihood of selective missing of debriefs in those cases where either the practice or the outcome were unfavourable. Presumably this bias did not vary over time and therefore is likely present at all time points in the data and thus although the magnitudes of complications might be underreported the trends are likely unaffected. Technical issues with the Qualtrics Offline mobile App used in Phase 2 were a potential hindrance to data collection and therefore also may have an unknown impact on the results. The mobile App was initially designed in such a fashion that it could store data offline and whenever connectivity was restored it could push the data off the phone into a web‐based cloud. Although this worked nicely during a small initial pilot with a sample of ten NMMs, technical issues were continually reported by Phase 2 mentors and thus a platform migration to ODK was implemented after 7 months. However, since two mentors were assigned to each facility, both of whom had a smartphone with the mobile App loaded this issue is not perceived to have prevented entry of a significant amount of data since if errors were encountered on one phone they could try on the other. Finally, although actual neonatal and maternal clinical outcomes would be the most rigorous way to assess the impact of the mobile debrief App, the unreliable birth and discharge registry data made this impossible in this context.

## 5 ETHICS APPROVAL AND CONSENT TO PARTICIPATE

All participants provided written consent for the use of video simulation data in an aggregated analysis. Ethics approval was granted from the institutional review boards of the University of California San Francisco (14‐15446) and the Indian Institute of Health Management Research.

## 6 CONCLUSION

In conclusion, we found that a mobile App is an acceptable job aid in a nurse‐mentoring programme and that it can be used to monitor changes in medical management, teamwork and supply availability over time. Further research could help determine if use of a mobile App improves debriefing skill and if debriefing itself has a positive impact on maternal and newborn clinical outcomes.

## CONFLICT OF INTEREST

Dilys Walker and Susanna Cohen are founding members of PRONTO International and sit on its board of directors. None of the other authors have any conflicts of interest to declare.
